# Comparison of Serum Trace Nutrient Concentrations in Epileptics Compared to Healthy Dogs

**DOI:** 10.3389/fvets.2019.00467

**Published:** 2019-12-19

**Authors:** Samantha Vitale, Devon Wallis Hague, Kari Foss, Maria Cattai de Godoy, Laura E. Selmic

**Affiliations:** ^1^Department of Veterinary Clinical Medicine, University of Illinois, Urbana, IL, United States; ^2^Department of Animal Sciences, University of Illinois, Urbana, IL, United States; ^3^Department of Veterinary Clinical Sciences, The Ohio State University, Columbus, OH, United States

**Keywords:** epilepsy, idiopathic epilepsy, copper, selenium, zinc, manganese, trace nutrients

## Abstract

Idiopathic epilepsy (IE) is a common cause of seizures in dogs. There are several investigations regarding serum concentrations of trace nutrients, including copper, selenium, zinc, manganese, and iron in human epileptics and animal models. However, research of this nature in dogs with epilepsy is lacking. The purpose of this prospective study was to compare serum concentrations of several trace nutrients in healthy dogs to dogs with idiopathic epilepsy. Healthy client-owned dogs (*n* = 50) and dogs with IE (*n* = 92) were enrolled and blood samples were collected for trace nutrient analysis. Epileptics were subdivided into three groups: controlled: *n* = 27, uncontrolled: *n* = 42, and untreated: *n* = 23. Serum was evaluated for concentrations of copper, selenium, zinc, cobalt, manganese, molybdenum, and iron using inductively coupled plasma mass spectroscopy. Uncontrolled epileptics had significantly higher manganese concentrations compared to normal dogs (*p* = 0.007). Untreated epileptics had higher iron levels than the other three groups (*p* = 0.04). Significantly higher levels of copper (*p* < 0.0001) were found in controlled and uncontrolled epileptics compared to normal or untreated dogs. Significantly higher levels of molybdenum (*p* = 0.01) were found in controlled epileptics compared to normal or untreated epileptics. Uncontrolled and controlled epileptics had significantly higher levels of selenium (*p* = 0.0003) vs. normal dogs, and uncontrolled epileptics had higher levels of zinc (*p* = 0.0002) than normal and untreated dogs. The significant difference in serum concentrations of several trace nutrients (manganese, selenium, and zinc) may suggest a role for these nutrients in the pathophysiology and/or treatment of epilepsy. Additionally, these results suggest that anti-convulsant therapy may affect copper and molybdenum metabolism.

## Introduction

Idiopathic epilepsy affects 0.5–1% of the canine population, and it has been reported in almost every breed (1). A genetic origin is presumed in some breeds and known in others (2, 3). Since little is known about the pathogenesis of this disease, there is no cure, and life-long management is limited to anti-convulsant therapy. Unfortunately, ~30% of these patients are considered refractory to one or more anti-convulsant medications (4). When multimodal anti-convulsant therapy fails to adequately control a patient's seizures, veterinarians are left without many alternatives, and euthanasia is often considered. The median survival time of patients after diagnosis of epilepsy (accounting for both euthanasia and natural death) was only 2.3 years in one study (5). As such, there is a constant search for complementary treatment options, ranging from vagal nerve stimulation to homeopathic supplementation (6).

Several investigations regarding the serum concentrations of trace nutrients, including copper, selenium, zinc, manganese, and iron in human epileptics and animal models have been performed. These studies suggest a correlation between low serum copper, manganese, and selenium levels in humans with epilepsy. In one study, serum copper levels were shown to be lower in children with idiopathic epilepsy who were not on anti-convulsant medication (7). A similar study measured selenium, zinc, and copper levels in children with intractable idiopathic epilepsy who were on at least two anti-convulsants. The epileptic children had lower serum selenium levels than the control group, however, there was no significant difference in their serum copper levels (8). Another study by Tutor-Crespo et al. suggests the differing results regarding copper levels could be explained by anti-convulsant therapy. Certain anti-convulsants, including phenobarbital, increase the oxidation of ceruloplasmin, consequently elevating serum ceruloplasmin and copper levels (9). A 2014 study evaluated epileptics of all ages and varying levels of seizure control, all of whom were on two or more anti-convulsants. This study showed a significantly higher serum copper level in epileptics compared to controls. The epileptic group was further broken down into poorly and well-controlled groups. There was no difference between copper levels in the poorly controlled and well-controlled epileptics (10). Manganese whole blood levels have also been reported to be lower in epileptics than controls, in both humans and experimental rodent models (11). To the author's knowledge, there are no current studies evaluating trace nutrient concentrations in epileptic dogs. We performed a pilot study in 2015 evaluating a total of 15 dogs, 7 normal dogs and 8 dogs with idiopathic epilepsy. Serum trace nutrient concentrations of copper, selenium, manganese, zinc, copper, molybdenum, and iron were evaluated, and significantly higher levels of manganese (*P* = 0.017), selenium (*p* = 0.018), and copper (*p* = 0.006) were found in the epileptic dogs. Given these findings, we elected to increase our sample size and subdivide our epileptic population further to better understand the significance of these data.

The purpose of this prospective study was to evaluate trace nutrient concentrations in the serum of canine epileptic patients compared to their healthy counterparts. Based on the results of our pilot study, we hypothesized that epileptic dogs would have higher serum selenium and manganese concentrations than normal dogs; epileptic dogs on anti-convulsant medications would have higher levels of copper than normal dogs or untreated epileptic dogs; and the untreated epileptic group would have lower serum copper than normal dogs.

## Materials and Methods

### Animals

Dogs presenting to the University of Illinois Veterinary Teaching Hospital for treatment of seizures were recruited for this study. In order to increase the number of controlled and untreated epileptics, patients were also recruited from local general practices. Additionally, 50 healthy adult dogs (at least 1 year of age) of any breed were recruited as part of the control population. Dogs were considered eligible for study enrollment in the epileptic population if they had a diagnosis of idiopathic epilepsy. For the purpose of this study, idiopathic epilepsy was defined as the first seizure occurring between 6 months-6 years of age, a normal neurologic exam (performed by a board-certified neurologist or neurology resident) in between seizures, and a complete blood count (CBC) and serum chemistry that did not reveal any clinically significant abnormalities. Brain magnetic resonance imaging (MRI) and cerebrospinal fluid (CSF) analysis were not required for inclusion in the study; however, if performed, patients were excluded if any abnormalities were noted on brain MRI or if there was evidence of inflammation on CSF analysis. Abnormal CSF was considered to be a total nucleated cell count of >5 cells/mm^3^ or protein >25 mg/dL (cisternal tap) or >45 mg/dL (lumbar tap), and/or abnormal interpretation by a clinical pathologist. Epileptic patients were also excluded if there were notable abnormalities on the CBC and serum chemistry or if progression of neurologic signs occurred in between seizures. Normal dogs were excluded from the study if there were notable abnormalities on the CBC and chemistry or if they developed seizures or other neurologic signs before the completion of the study. Informed client consent was obtained in writing prior to inclusion, and this study was approved by the Institutional Animal Care and Use Committee of the University of Illinois. Epileptic patients were divided into three subgroups:

1. Untreated Epileptics: Dogs that had not received anti-convulsant therapy in the past 30 days prior to enrollment, other than diazepam as needed to break a seizure on presentation to the ER.
2. Controlled Epileptics: Dogs that were treated with one or more anti-convulsants and had fewer than or equal to 1 seizure every 2 months with no episodes of cluster seizures or status epilepticus after starting medication.
3. Uncontrolled Epileptics: Dogs that were treated with one or more anti-convulsants and were having >1 seizure every 2 months, cluster seizures, or status epilepticus despite therapy.

### Sample Collection

All dogs had a physical and neurologic examination performed by a board-certified neurologist and/or neurology resident. Owners of all dogs filled out a standardized history form regarding seizure frequency, duration of seizures, age at first seizure, degree of seizure control, medications and dosages. A diet history was also collected for all patients using the World Small Animal Veterinary Association (WSAVA)^1^ short diet history form. All dogs had blood samples collected for complete blood count, serum chemistry profile, and trace nutrient analysis. Blood for trace nutrient analysis was collected into a royal blue top tube^2^, which contains no additives, and is specifically purposed for trace nutrient analysis as it does not bind any elements. The blood was clotted for 10–15 min and centrifuged at 3600 RPM for 10 min. The serum was collected and stored in a glass tube in a −20°F (−28.9°C) freezer for temporary storage, then transferred to a −80°F (−62.2°C) freezer within a week for long-term storage prior to analysis. Trace nutrient analysis (total element) for copper (Cu), selenium (Se), zinc (Zn), cobalt (Co), manganese (Mn), molybdenum (Mo), and iron (Fe) was performed by inductively coupled plasma mass spectroscopy at the Michigan State University Diagnostic Center for Population and Animal Health. The detection level for each element is reported by the lab as follows: Cu: 0.02 μg/mL, Se: 0.2 ng/mL, Co: 0.1 ng/mL, Mn: 0.5 ng/mL, Mo: 0.5 ng/mL, Fe: 7 mg/dL.

### Statistical Analysis

Continuous variables were assessed for normality using skewness, kurtosis, and Shapiro Wilk tests. If data were normally distributed, the mean and standard deviation were used to summarize. If the data were non-normally distributed these were summarized using median and interquartile range. Baseline characteristics of groups including sex, age, and breed were compared using Fisher exact tests and Kruskal–Wallis tests.

Trace element concentration variables were log transformed for application of parametric statistical analysis and to decrease effects of outliers. For evaluation of differences in trace element concentrations in the epileptic vs. normal population student *t*-tests were used. For evaluation for effects of treatment on trace element concentrations, dogs were grouped as treated epileptics, untreated epileptics or normal. Welch's ANOVA was used with pairwise comparisons using Bonferroni *t*-tests. To evaluate for differences in trace element concentration with seizure control, dogs were grouped into epileptic controlled, uncontrolled, untreated and normal. Welch's ANOVA was used, with pairwise comparisons using Bonferroni *t*-tests.

Commercially available software^3^ was used for the statistical analysis and a *p* < 0.05 was considered statistically significant.

## Results

A total of 158 dogs were recruited for enrollment in this study. Sixteen patients were excluded (4 due to concurrent disease, 3 due to significant abnormalities on CBC or chemistry, 1 due to abnormal MRI findings after enrollment, 1 normal dog due to the development of seizures before the completion of the study, 5 due to loss of the samples, 1 due to a history of head trauma prior to the onset of seizures, and 1 due to correction of reported age at first seizure). Therefore, a total of 142 dogs were enrolled (50 normal dogs and 92 dogs with suspected or confirmed idiopathic epilepsy).

In the normal group, there were 21 spayed females, 28 castrated males, and 1 intact male. The ages of the normal dogs ranged from 1 to 12 years old, with a median of 5.3 years (Table 1). Breeds represented included: 4 Australian shepherds, 2 cattle dogs, 3 Labrador retrievers, 4 pit bull terriers, 21 mixed breed dogs, and one each of Airedale terrier, beagle, Border collie, Border terrier, boxer, bull mastiff, Chesapeake Bay retriever, German shepherd, golden retriever, Great Dane, Siberian husky, labradoodle, miniature Australian shepherd, Pembroke Welsh corgi, Samoyed, and Yorkshire terrier.

**Table 1:** Patient demographics by group.

**Group (number of dogs)**	**Spayed females**	**Intact females**	**Castrated males**	**Intact males**	**Median age (years)**
Normal (50)	21	0	28	1	5.3 (1–12)
Controlled epileptic (27)	10	0	14	3	6.8 (2–14.5)
Uncontrolled epileptic (42)	12	0	26	4	4 (1-10.8)
Untreated epileptic (23)	6	1	15	1	4.5 (1–12)

The epileptic dogs were divided into 3 subgroups: controlled epileptics (12), uncontrolled epileptics (42), and untreated epileptics (13). In the controlled epileptic group, there were 10 spayed females, 14 castrated males, and 3 intact males. The ages of the controlled epileptics ranged from 2 to 14.5 years, with a median of 6.8 years (Table 1). Breeds represented included: 2 beagles, 2 cocker spaniels, 3 dachshunds, 6 mixed breed dogs, 2 English mastiffs, and one each of Akita, Bichon Frise, Border collie, chihuahua, English bulldog, goldendoodle, golden retriever, greyhound, Labrador retriever, Maltese, miniature schnauzer, and Rottweiler.

In the uncontrolled epileptic group, there were 12 spayed females, 26 castrated males, and 4 intact males. Their ages ranged from 1 to 10.8 years with a median of 4 years (Table 1). Breeds included: 2 English bulldogs, 2 English springer spaniels, 2 German shepherds, 4 golden retrievers, 2 German shorthaired pointers, 2 Labrador retrievers, 15 mixed breed dogs, and one each of Australian shepherd, beagle, bloodhound, chihuahua, chow chow, greyhound, Malt-a-poo, English mastiff, pit bull terrier, pug, Shih tzu, Siberian husky, and Saint Bernard breeds.

In the untreated epileptic group, there were 6 spayed females, 1 intact female, 15 castrated males, and 1 intact male. Their ages ranged from 1 to 12 years with a median of 4.5 years (Table 1). Breeds represented included: 2 Golden retrievers, 7 mixed breed dogs, 2 Siberian huskies, and one each of the Alaskan malamute, Australian Shepherd, dachshund, English bulldog, French bulldog, German shorthaired pointer, goldendoodle, Great Pyrenees, Irish setter, Labrador retriever, miniature poodle, and miniature dachshund breeds.

There were no statistically significant differences in age, breed, or gender between groups.

A diet history was provided by the owners of 139/142 dogs (98.0%). There were a variety of diets represented, and most patients were fed treats and table food in addition to their main diet. All 139 of the patients enrolled were being primarily fed a commercially available diet (dry and/or canned), and none of the dogs were being fed an entirely home cooked or raw diet.

In the controlled epileptic group, 17/27 dogs (63.0%) were taking only one anti-convulsant (6/17 levetiracetam only, 8/17 phenobarbital only, 3/17 zonisamide only). Eight out of 27 dogs (29.6%) were taking two anti-convulsants, and 2/27 (7.4%) were taking three anti-convulsants. In the uncontrolled epileptic group, 12/42 dogs (28.5%) were taking only one anti-convulsant drug (5/12 levetiracetam only and 7/12 phenobarbital only), 16/42 dogs (38.0%) were taking two anti-convulsants, and 14/42 dogs (33.3%) were taking three or more anti-convulsants (Supplementary Table 1). Dosage information was recorded based on medical records and owner recollection, but was not available for all cases.

All normal dogs had a neurologic exam, complete blood count, and serum chemistry performed with no clinically significant findings. All epileptic patients had a normal neurologic exam and serum chemistry performed. All but 2 epileptic patients had a complete blood count performed as 2 controlled epileptics did not have adequate sample for a CBC. One normal dog had a brain MRI for reasons unrelated to any neurologic disorder, which was normal. Of the controlled epileptics 6 had an MRI and CSF analysis. In the uncontrolled epileptic group, 2 had an MRI only, 11 had an MRI and CSF analysis, 2 had an MRI, CSF analysis, and necropsy, and 1 had a necropsy only. Of the untreated epileptic group, 8 dogs had an MRI and CSF analysis. All of the patients who underwent advanced imaging ± CSF analysis had unremarkable MRI and CSF analysis consistent with idiopathic epilepsy. No gross or histopathologic abnormalities were found in the brains of any of the patients who had necropsies.

There were differences in several of the trace nutrients between groups (Table 2). There were no significant differences in the serum cobalt levels between groups. There were also no significant differences in iron levels between normal and epileptic dogs. However, when dividing the epileptics into the subgroups, the untreated epileptic dogs had significantly higher serum iron levels than the other three groups (*p* = 0.04). Additionally, overall, no significant differences in molybdenum levels were found between the normal and epileptic groups. But again, when divided, the controlled epileptic dogs had significantly higher molybdenum levels than either the untreated epileptic dogs or the normal dogs (*p* = 0.01) (Table 3).

**Table 2:** Trace nutrient concentrations in normal dogs (*n* = 50) compared to all epileptic dogs as a whole (*n* = 92).

**Trace nutrients in serum**	**Category**	**Normal (*n* = 50)**	**Epileptic (*n* = 92)**	***p*-value**
Cobalt (ng/ml)	Median, IQR	0.30, 0.15	0.31, 0.17	0.57
Copper (μg/ml)	Median, IQR	0.45, 0.09	0.53, 018	<0.0001
Iron (μg/dL)	Median, IQR	162.50, 51.00	165.00, 65.00	0.3
Manganese (ng/ml)	Median, IQR	3.15, 1.30	4.10, 1.90	0.0006
Molybdenum (ng/ml)	Median, IQR	8.45, 7.80	10.10, 9.50	0.13
Selenium (ng/ml)	Median, IQR	300.50, 52.00	336.00, 66.00	<0.0001
Zinc (μg/ml)	Median, IQR	0.74, 0.13	0.85, 0.33	0.0007

*Significance was defined as a p < 0.05. IQR, interquartile range*.

**Table 3:** Trace nutrient concentrations in normal dogs (*n* = 50) compared to the individual subgroups of epileptic dogs.

**Trace nutrients in serum**	**Category**	**Normal (*n* = 50)**	**Epileptic untreated (*n* = 23)**	**Epileptic controlled (*n* = 27)**	**Epileptic uncontrolled (*n* = 42)**	***p*-value**
Cobalt (ng/ml)	Median, IQR	0.35, 0.15	0.35, 0.19	0.30, 0.21	0.28, 0.16	0.75
Copper (μg/ml)	Median, IQR	0.45, 0.09	0.46, 0.09	0.63, 0.19	0.55, 0.15	<0.0001
Iron (μg/dL)	Median, IQR	162.50, 51.00	190.00, 56.00	152.00, 52.00	157.00, 72.00	0.04
Manganese (ng/ml)	Median, IQR	3.15, 1.30	3.40, 3.70	4.10, 1.60	4.20, 2.10	0.007
Molybdenum (ng/ml)	Median, IQR	8.45, 7.80	8.80, 6.80	14.20, 7.40	8.60, 11.10	0.01
Selenium (ng/ml)	Median, IQR	300.50, 52.00	320.00, 56.00	343.00, 71.00	343.00, 71.00	0.0003
Zinc (μg/ml)	Median, IQR	0.74, 0.13	0.70, 0.12	0.85, 0.25	0.94, 0.23	0.0002

**Figure 1:**
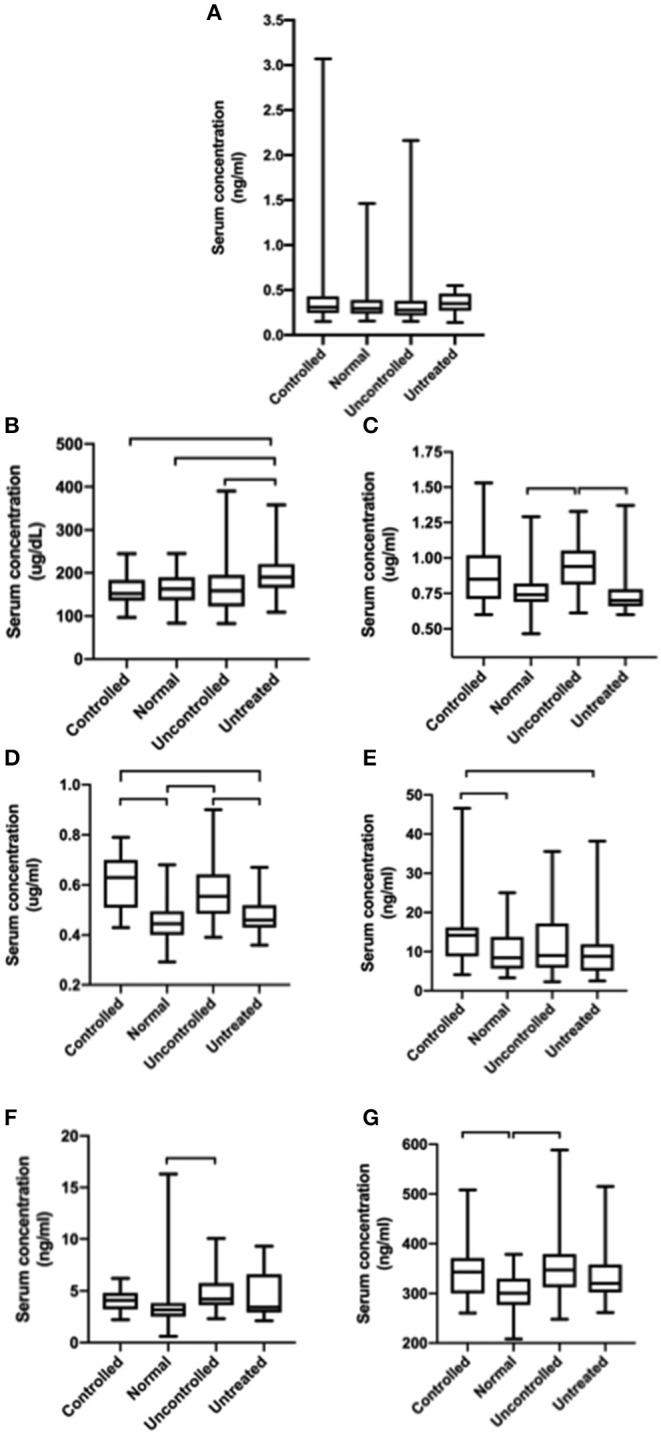
Box and Whisker plots show the distribution of each trace nutrient between epileptic subgroups and normal dogs. Statistically significant differences between groups are highlighted using brackets. **(A)** Cobalt, **(B)** Iron, **(C)** Zinc, **(D)** Copper, **(E)** Molybdenum, **(F)** Manganese, and **(G)** Selenium.

Copper (*p* < 0.0001), manganese (*p* = 0.007), selenium (*p* = 0.0003), and zinc (*p* = 0.0002) levels were all significantly increased in epileptic dogs compared to normal dogs. When divided into epileptic subgroups, copper levels were significantly higher in controlled and uncontrolled epileptic dogs compared to untreated epileptic dogs and normal dogs. Manganese levels were significantly higher in uncontrolled epileptic dogs compared to normal dogs. There were significantly higher levels of selenium in controlled and uncontrolled epileptic dogs compared to normal dogs. Finally, the uncontrolled epileptic dogs had significantly higher zinc levels than untreated epileptic dogs and normal dogs (Figure 1).

The lab reported that 19/142 samples (13.4%) were hemolyzed (5/50 normals, 4/42 uncontrolled epileptics, 9/27 controlled epileptics, and 2/23 untreated epileptics). Red blood cell hemolysis has the potential to release intracellular elements, such as selenium, manganese, and zinc from the red blood cell into the serum, therefore falsely elevating the serum concentrations of these elements (14). Therefore, the comparisons were re-analyzed with these 19 dogs excluded for selenium, manganese, and zinc. Exclusion of these 19 dogs did not alter the significance of the findings.

Of the treated epileptic dogs, 49/69 dogs (71%) were on phenobarbital. The copper and molybdenum levels of dogs on phenobarbital (as a monotherapy or in conjunction with other medications) and dogs being treated with anti-convulsants besides phenobarbital were independently compared to both untreated epileptics and normal dogs. There was a significant difference in the serum copper levels between the dogs that were on phenobarbital and the untreated and normal dogs (*p* < 0.0001). There was also a significant difference between the dogs on anti-convulsants other than phenobarbital and normal dogs, but no significant difference between non-phenobarbital treated epileptics and untreated epileptics. There were no significant pairwise comparisons between the molybdenum levels of these groups (*p* = 0.38).

## Discussion

This study showed several differences in trace nutrient levels between normal and epileptic dogs, as well as within the epileptic subgroups. Copper (Cu) and molybdenum (Mo) serum levels were higher in dogs receiving anti-convulsant therapy than untreated epileptic dogs or normal dogs, which suggests that anti-convulsant medications may affect the levels of these nutrients. Manganese (Mn), selenium (Se), and zinc (Zn) serum levels were also higher in uncontrolled epileptic dogs than normal dogs, which suggests a possible role in the pathophysiology of epilepsy, although it is not clear whether these elevations are the cause of poor seizure control or an effect of the seizures themselves.

Patients on anti-convulsant medications (both controlled and uncontrolled), had significantly higher copper levels than normal dogs or epileptics that were not yet treated, similar to what is reported in people. However, there were not significant differences in the copper levels of controlled vs. uncontrolled epileptic dogs or in untreated dogs vs. normal dogs. This suggests a possible influence of certain anti-convulsant medications on copper metabolism. This is similar to several human reports that certain anti-convulsant medications, including phenobarbital, phenytoin, and carbamazepine can increase serum copper and ceruloplasmin levels (9).

Treated epileptics in this study were on a variety of medications, including phenobarbital, levetiracetam, potassium bromide, zonisamide, and gabapentin and several patients were on multiple anti-convulsants. Of those patients, 49/69 (71%) were on phenobarbital regularly, which is known to induce hepatic enzymes (15). When comparing the copper levels of dogs who were on phenobarbital to untreated epileptic dogs, there was a significant difference between the group of treated epileptics on phenobarbital and the untreated dogs, but not between the treated epileptics that were not on phenobarbital and the untreated dogs. Therefore, it is likely that the serum copper elevations in this study were a result of increased activity of hepatic enzymes, likely secondary to phenobarbital, and is unlikely to be associated with seizure control.

Controlled epileptic dogs also had significantly higher molybdenum levels compared to untreated epileptics or normal dogs. It is possible that the anti-convulsant medications used affect molybdenum metabolism as well. However, it is interesting that there is a similar pattern of copper and molybdenum elevation, given that molybdenum is known to antagonize copper absorption in ruminants (16). There is very little information in the literature regarding molybdenum metabolism in small animals. A recent study evaluated the effect of NDMA induced hepatic fibrosis in rats on trace nutrient concentrations in the serum and the liver. Rats had higher serum copper and molybdenum levels during the oxidative stress of developing hepatic fibrosis. However, the study was unable to determine the cause of the serum molybdenum levels, and speculated that some process, potentially interaction with the other nutrients, resulted in mobilization of molybdenum from the liver (17). In order to better understand the effect of anti-convulsant medications on molybdenum in small animals, further research on molybdenum metabolism and nutritional requirements is warranted.

Manganese levels were significantly higher in uncontrolled epileptics compared to normal dogs, which suggests that poor seizure control may be related to elevated manganese levels. This differs from the findings in several human and rodent studies, where blood and or brain levels of manganese were significantly lower in epileptics than control patients. However, these studies do not identify the cause of the lower manganese levels (11). Manganese toxicity in humans is associated with significant neurologic symptoms, including behavior change, compulsive behavior, dystonia, and tremors^4^. Additionally, there are case reports of humans with manganese toxicity who developed seizures (18). While we have no reason to believe that any our patients were suffering from manganese toxicity, it would be worthwhile to determine appropriate reference ranges for serum manganese in dogs to evaluate uncontrolled epileptics or patients with a presumed neurotoxic cause for seizures. Finally, there is evidence in humans that hair manganese levels may be a more accurate reflection of the body's true manganese exposure, and that blood or serum manganese levels may fluctuate and be less reliable, which may also be the case in our study patients (19).

Both uncontrolled and controlled epileptics had significantly higher selenium levels than normal patients, but there was no difference between untreated epileptics and normal dogs. This also differs from the human literature (8), in which uncontrolled epileptics were found to have lower serum selenium levels than non-epileptic patients (8). A 2016 paper showed that epileptics on carbamazepine and valproic acid had lower serum selenium levels than patients on other drugs (20). It is possible that the results of the Seven et al. study were influenced by the anti-convulsant medications prescribed, and since none of our patients take these two medications, we did not appreciate the same effect (20). Furthermore, there are reports of selenium deficiency being associated with intractable seizures in children, who improved with selenium supplementation (21). The published reference range for serum selenium in adult dogs is 200–300 ng/mL. None of the dogs in the current study had a selenium level below the reference range. In fact, 85 patients (25/50 normal dogs, 18/23 untreated dogs, 22/42 uncontrolled dogs, and 20/27 controlled dogs) had selenium levels >300. While selenium deficiency may be just as likely to cause seizures in dogs as humans, dogs are more likely to eat a commercial, balanced diet, and none of the patients in this study were exhibiting selenium deficiency.

Finally, uncontrolled epileptics had significantly higher zinc levels than untreated epileptics or normal patients. Zinc binds to the delta subunit of extrasynaptic GABA-A receptors in the brain (22). This is also the preferred binding site of endogenous neurosteroids, which are a topic of epilepsy research in humans due to their anti-convulsant effects (23). Of the free (non-protein bound) zinc in the brain, most of it is contained within synaptic vesicles, primarily in the hippocampus (a highly epileptogenic region) and the olfactory bulb (13). In a mouse model of kindling induced epilepsy, zinc was shown to significantly decrease the anti-convulsant effects of a synthetic neurosteroid, ganaxolone, when administered by infusion into the hippocampus (22). The results of this study and the significantly higher zinc levels in our uncontrolled epileptics suggest further investigation regarding the role of zinc in the pathophysiology of epilepsy.

There are a few case reports and case-controlled studies evaluating trace mineral supplementation in people with epilepsy, indicating that there may be opportunities for novel treatments in epileptic patients. Recently, a study showed that pediatric patients with intractable epilepsy had low serum levels of zinc and selenium. In those patients, zinc supplementation significantly reduced seizure frequency and when the zinc supplement was discontinued, the seizure activity recurred (24). There is also some evidence that zinc supplementation decreases seizure activity in experimental seizure models in rats (25). Additionally, there are a few case reports in human epileptics documenting a selenium deficiency that improved with selenium supplementation (12, 21, 26). In this study population, however, the poorly controlled epileptics had higher levels of zinc, selenium, and manganese, so further studies regarding supplementation in these patients would be expected to be unrewarding. At this time, we are unsure whether elevations in any of these nutrients predispose seizures in genetically prone patients or whether the elevated levels are a result of the seizures themselves.

It is also unknown if the nutrient alterations are a part of the seizure pathogenesis or an effect. Further studies measuring serum concentrations of zinc, selenium, and manganese in uncontrolled epileptics within 24 h of a seizure and again after a seizure-free period may help elucidate whether the elevated levels are secondary to seizures (i.e., a neuroprotective effect) vs. involved in the pathogenesis. We recorded the time from the most recent seizure for each study patient, however, since owner recollection is required to determine the exact time, we do not have reliable data for all enrolled patients. Additionally, these study participants were presenting either for seizure management or routine follow up, so we were unable to standardize the time from the last seizure in this study.

Our patient population had drastically different types of diets than people, and typically dogs that eat a balanced dog food do not suffer from dietary deficiencies. Many of the human studies referenced above took place in Middle East or Asia, which may have variations in elemental soil content and dietary differences compared to the United States. To the author's knowledge, no studies regarding trace nutrient concentrations in human epileptics in the United States have been performed. Diet histories were obtained for all but three patients enrolled, however, given the extreme variability in diets fed and the inability to control for diet in this study, we did not perform statistical analysis. All owners who provided a diet history stated that their dogs were fed primarily a commercially available dog food rather than a home-cooked or raw diet. A recent study analyzed 49 commercially available dry dog foods for concentrations of chromium, nickel, molybdenum, silica, and aluminum, and found that on average, the diets contained much higher concentrations of all of these elements than the average human consumes in a day (27). Unfortunately, only one of the elements that we studied was assessed in this study. Further research would be required to evaluate the concentrations of other nutrients, such as copper, selenium, and zinc to determine if they are higher in pet food than the average human diet and may be contributing to the different patterns in our patients than humans. Further studies could involve repeating this analysis while patients are being fed a consistent diet to see if diet plays a role in trace nutrient levels and seizure control.

There were several limitations to this study. First, trace nutrient measurements were performed on serum, rather than CSF, and may not accurately reflect the influence of these nutrients on central nervous system disease. Collection of CSF is more invasive than sampling blood. Therefore, it is likely fewer clients would have been willing to subject a healthy patient to anesthesia and CSF collection. Additionally, serum levels of trace nutrients may reflect a transient value, and analysis of liver, kidney, hair, or nail concentrations may be a more accurate reflection of the body's true stores. At this time, there are no published references ranges in dogs for cobalt, manganese, and molybdenum. Additionally, given that we could not control or predict when eligible dogs would present to the Veterinary Teaching Hospital, we were unable to regulate timing of feeding in relationship to time of blood draw, which could potentially influence blood levels of certain nutrients. An additional limitation exists regarding the interpretation of the cause of our observed selenium elevations. While none of the dogs in our study were on carbamazepine or valproic acid, it is possible that one of the anti-convulsant drugs they were receiving could influence selenium metabolism. Given the wide variety of numbers and types of anti-convulsant medications that our controlled and uncontrolled epileptics were receiving, it would be challenging to determine whether a significant association exists with one individual drug. This would be worthwhile to investigate in a future prospective study involving patients on standardized regimen of anti-convulsants. Another limitation of this study was the low number of untreated epileptics. As a tertiary referral institution, most of the epileptic patients presenting were already on anti-convulsant therapy before seeing a specialist. In an effort to increase the number of untreated and well-controlled patients, we recruited cases from the referring veterinarians in the surrounding metropolitan area.

## Conclusion

In summary, epileptics receiving anti-convuslant therapy had higher levels of copper and molybdenum, likely due to the effects of the anti-convulsants themselves. Manganese and zinc levels were higher in uncontrolled epileptics than normal dogs, and selenium levels were higher in both controlled and uncontrolled epileptics than normal dogs. The potential role of manganese, selenium, and zinc in the pathophysiology of epilepsy cannot be elucidated from a study of this nature; however, these findings warrant further investigation. The results of this study do not justify restriction or supplementation of any trace nutrient in epileptic patients at this time. Both toxicity and deficiency of all trace nutrients can have detrimental systemic and neurologic effects, and dietary modification of trace nutrients is not recommended without further investigation. Ultimately, the results of this study shed additional light on the differences between epileptic and non-epileptic dogs, and open the door for additional investigation regarding the significance of these differences.

## Data Availability Statement

The datasets generated for this study are available on request to the corresponding author.

## Ethics Statement

The animal study was reviewed and approved by Institutional Animal Care and Use Committee at the University of Illinois at Urbana-Champaign. Written informed consent was obtained from the owners for the participation of their animals in this study.

## Author Contributions

SV: study design, sample collection, data interpretation, and manuscript writing. DH and KF: study design, data interpretation, and manuscript writing. MG: study design and data interpretation. LS: statistical analysis and manuscript writing.

### Conflict of Interest

The authors declare that the research was conducted in the absence of any commercial or financial relationships that could be construed as a potential conflict of interest.

## Footnotes

^1^https://www.wsava.org/WSAVA/media/Arpita-and-Emma-editorial/Diet-History-Form.pdf

^2^BD Vacutainer Trace Element Serum Blood Collection Tubes, Becton, Dickenson and Company, Franklin Lakes, NJ, USA.

^3^SAS Version 9.4 for Windows, SAS Institute Inc., Cary, NC, USA.

^4^https://www.bioscience.org/2018/v23/af/4665/fulltext.php?bframe=PDF (accessed February 21, 2019).
