# Supplementary material for: Comparison of Serum Trace Nutrient Concentrations in Epileptics Compared to Healthy Dogs
Source: Front Vet Sci. 2019 Dec 19;6:467. doi: 10.3389/fvets.2019.00467 (PMC6930867; doi:10.3389/fvets.2019.00467)
Supplement: Supplementary file 1 [file Table_1.docx]

Supplementary Table (S1): Summary of anti-convulsants for each patient in the uncontrolled and controlled epileptic groups. KBr; potassium bromide.

| **Patient** | **Controlled or Uncontrolled** | **Anti-convulsant Medication** |
| --- | --- | --- |
| 1 | Uncontrolled | levetiracetam, phenobarbital, diazepam |
| 2 | Uncontrolled | phenobarbital, gabapentin |
| 3 | Uncontrolled | phenobarbital, levetiracetam |
| 4 | Uncontrolled | phenobarbital, levetiracetam |
| 5 | Uncontrolled | phenobarbital, zonisamide, diazepam |
| 6 | Uncontrolled | phenobarbital |
| 7 | Uncontrolled | phenobarbital, diazepam, levetiracetam, gabapentin |
| 8 | Uncontrolled | phenobarbital, KBr, levetiracetam |
| 9 | Uncontrolled | phenobarbital, KBr, levetiracetam |
| 10 | Uncontrolled | phenobarbital, levetiracetam |
| 11 | Uncontrolled | phenobarbital, levetiracetam |
| 12 | Uncontrolled | phenobarbital, KBr, levetiracetam |
| 13 | Uncontrolled | levetiracetam |
| 14 | Uncontrolled | phenobarbital |
| 15 | Uncontrolled | phenobarbital, KBr, levetiracetam, zonisamide |
| 16 | Uncontrolled | phenobarbital, levetiracetam |
| 17 | Uncontrolled | KBr, levetiracetam |
| 18 | Uncontrolled | phenobarbital, KBr |
| 19 | Uncontrolled | phenobarbital, levetiracetam |
| 20 | Uncontrolled | phenobarbital |
| 21 | Uncontrolled | phenobarbital, KBr, zonisamide, diazepam |
| 22 | Uncontrolled | levetiracetam |
| 23 | Uncontrolled | phenobarbital, levetiracetam, diazepam |
| 24 | Uncontrolled | levetiracetam |
| 25 | Uncontrolled | phenobarbital, KBr |
| 26 | Uncontrolled | phenobarbital, levetiracetam |
| 27 | Uncontrolled | phenobarbital, levetiracetam |
| 28 | Uncontrolled | phenobarbital, diazepam, levetiracetam, gabapentin |
| 29 | Uncontrolled | phenobarbital, KBr, diazepam |
| 30 | Uncontrolled | phenobarbital |
| 31 | Uncontrolled | phenobarbital |
| 32 | Uncontrolled | levetiracetam |
| 33 | Uncontrolled | KBr, levetiracetam |
| 34 | Uncontrolled | KBr, levetiracetam, zonisamide |
| 35 | Uncontrolled | phenobarbital, levetiracetam |
| 36 | Uncontrolled | phenobarbital, levetiracetam, KBr |
| 37 | Uncontrolled | phenobarbital, levetiracetam, diazepam |
| 38 | Uncontrolled | phenobarbital |
| 39 | Uncontrolled | levetiracetam |
| 40 | Uncontrolled | phenobarbital |
| 41 | Uncontrolled | phenobarbital, KBr |
| 42 | Uncontrolled | levetiracetam, zonisamide |
| 43 | Controlled | levetiracetam |
| 44 | Controlled | zonisamide |
| 45 | Controlled | phenobarbital, KBr |
| 46 | Controlled | phenobarbital, zonisamide |
| 47 | Controlled | phenobarbital |
| 48 | Controlled | zonisamide |
| 49 | Controlled | phenobarbital |
| 50 | Controlled | levetiracetam |
| 51 | Controlled | levetiracetam |
| 52 | Controlled | zonisamide |
| 53 | Controlled | phenobarbital, KBr |
| 54 | Controlled | phenobarbital |
| 55 | Controlled | levetiracetam |
| 56 | Controlled | phenobarbital |
| 57 | Controlled | phenobarbital, KBr |
| 58 | Controlled | phenobarbital, levetiracetam, diazepam |
| 59 | Controlled | phenobarbital |
| 60 | Controlled | zonisamide, levetiracetam |
| 61 | Controlled | levetiracetam |
| 62 | Controlled | phenobarbital, zonisamide |
| 63 | Controlled | phenobarbital |
| 64 | Controlled | levetiracetam |
| 65 | Controlled | phenobarbital, KBr |
| 66 | Controlled | phenobarbital, levetiracetam |
| 67 | Controlled | phenobarbital |
| 68 | Controlled | phenobarbital |
| 69 | Controlled | phenobarbital, KBr, levetiracetam |
